# Supplementary material for: Mendelian segregation and high recombination rates facilitate genetic analyses in Cryptosporidium parvum
Source: bioRxiv. 2024 Feb 2:2024.02.02.578536. Preprint. [Version 1] doi: 10.1101/2024.02.02.578536 (PMC10862819; doi:10.1101/2024.02.02.578536)
Supplement: Supplement 1 [file NIHPP2024.02.02.578536v1-supplement-1.pdf]

Supporting Materials:

S1 Fig. Yellow oocyst populations vary in GFP and mCherry expression in vitro and in vivo on different days of infection.

S2 Fig. The ABC gene was successfully tagged in *Cryptosporidium parvum*.

S3 Fig. The Enolase gene was successfully tagged in *Cryptosporidium parvum.*

Movie S1. Progeny from outcrossed oocysts infect HCT-8 cells and display a tetratype segregation pattern.

S1 Table. Key reagents and resources used in the experiments.

S2 Table. All primers used for the construction of the ABC-HA-CFP and Enolase-HA transgenic parasites.
